# Computational complexity drives sustained deliberation

**DOI:** 10.1038/s41593-023-01307-6

**Published:** 2023-04-24

**Authors:** Tao Hong, William R. Stauffer

**Affiliations:** 1 Department of Neurobiology, University of Pittsburgh, Pittsburgh, PA USA; 2 Program in Neural Computation, Carnegie Mellon University, Pittsburgh, PA USA; 3 Center for the Neural Basis of Cognition, Pittsburgh, PA USA

**Keywords:** Problem solving, Reward, Decision

## Abstract

Economic deliberations are slow, effortful and intentional searches for solutions to difficult economic problems. Although such deliberations are critical for making sound decisions, the underlying reasoning strategies and neurobiological substrates remain poorly understood. Here two nonhuman primates performed a combinatorial optimization task to identify valuable subsets and satisfy predefined constraints. Their behavior revealed evidence of combinatorial reasoning—when low-complexity algorithms that consider items one at a time provided optimal solutions, the animals adopted low-complexity reasoning strategies. When greater computational resources were required, the animals approximated high-complexity algorithms that search for optimal combinations. The deliberation times reflected the demands created by computational complexity—high-complexity algorithms require more operations and, concomitantly, the animals deliberated for longer durations. Recurrent neural networks that mimicked low- and high-complexity algorithms also reflected the behavioral deliberation times and were used to reveal algorithm-specific computations that support economic deliberation. These findings reveal evidence for algorithm-based reasoning and establish a paradigm for studying the neurophysiological basis for sustained deliberation.

## Abstract (web summary)

The authors develop a reward optimization framework to study sustained deliberation in nonhuman primates. As the computational complexity increased, animals deliberated longer and applied more complex reasoning strategies to optimize rewards.

## Main

Difficult economic decisions—whether to buy a house or not, what to study at school or how to invest for retirement—require slow, effortful and intentional consideration^1^. Decision-makers must determine the best outcomes by considering combinations of economic factors, including immediate needs, available resources, environmental constraints and future consequences. These combinatorial considerations generate substantial computational demands and render the finding of satisfactory solutions to a hard problem. Economic deliberation is, therefore, a cognitive process of identifying subjectively optimal solutions to hard economic problems. This mode of slow and effortful thinking is rarely evoked with traditional neuroeconomic paradigms. Even when choosing between items with similar values, a context that is considered to drive hard economic choices, both human and nonhuman primate (NHP) neuronal and behavioral responses evolve in hundreds of milliseconds^2,3^. In contrast to standard neuroeconomic tasks, combinatorial optimization problems require extended deliberations. These problems can be designed to reflect the combinatorial nature of difficult economic decisions, as defined above, and possess a normative scale of difficulty—computational complexity. Recent studies of human decision-makers have shown that computational complexity modulates effort, response times (RTs) and performance^4,5^. Thus, combinatorial optimization tasks have the capacity to model difficult economic decisions, and to reveal the mental algorithms for economic deliberation.

Computational difficulty, or complexity, is manifested in numerous routine tasks, including scheduling, route planning and, as mentioned above, economic decision making^6^. Therefore, understanding how the brain manages computational complexity is vital for building computationally plausible models of cognition and behavior^7,8^. Computational complexity theory is a branch of computer science that focuses on characterizing the difficulty of solving computational problems^9^, defining the complexity of individual problems^10^ and developing efficient algorithms to approximate optimal solutions to computationally intractable optimization problems^11–13^. Concepts from computational complexity theory can be directly applied to the computations that brains must accomplish. For example, one well-known combinatorial optimization problem, the traveling salesman problem, has proven relevant to the analysis of spatial cognition and the diagnoses of cognitive impairments^14–18^. This highlights the fundamental link between computation and cognition^19,20^, and emphasizes the fact that linking the brain to behavior requires an understanding of the computational principles of thought.

The knapsack problem is a nonspatial combinatorial optimization problem that asks agents to identify valuable combinations that satisfy constraints^21^. Decades of computer science research have generated a number of efficient algorithms that approximate optimal solutions^11–13^. Recent studies of human participants have shown that computational difficulty impacts performance, and that the knapsack problem is a generally useful framework to define and study complex decision making^4,5^. Here we devised an NHP ‘knapsack task’, based on the eponymous problem^21^, with the goal of promoting temporally extended economic deliberations. We collected behavioral data from two animals performing the knapsack task. We used several algorithmic parameters, including a clustering parameter, *k*^4,12^, and a thresholding parameter, *t*^13^, to quantify the complexity of the task. We classified behavioral solutions according to ‘low-complexity’ combinatorial algorithms that consider items one at a time, such as the greedy algorithm^11^, or ‘high-complexity’ combinatorial algorithms that search for valuable combinations, such as the Sahni-*k* and Johnson-*t* algorithms^12,13^. We demonstrated that computational complexity influences reasoning strategies and revealed the dominant algorithms the animals used to optimize rewards.

## Results

### NHPs sought to optimize outcomes in the knapsack task

**Fig. 1:**
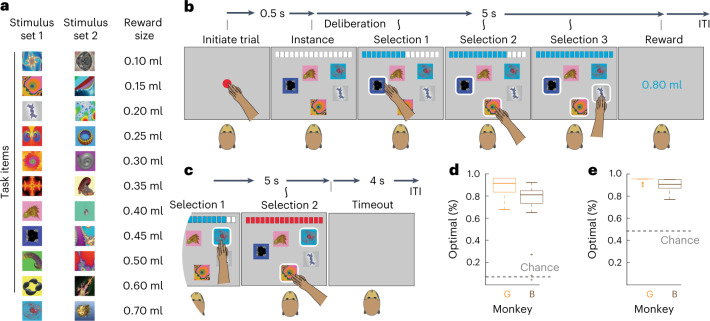
Knapsack task and basic optimizing behaviors. **a**, List of fractal images that symbolized the items in stimulus set 1 (left column), stimulus set 2 (middle column) and the items’ reward sizes (right column). **b**, Schematic representation of the knapsack task. The animals initiated the knapsack trials by touching a red central target. After initiation, an instance was displayed and remained on the screen for 5 s. During those 5 s, the number and identity of the selections were determined by the animal. When they selected an item, it was highlighted, it could not be deselected and the volume associated with the selection was added to the virtual knapsack at the top of the screen. If the sum of the items was less than or equal to 0.8 ml, then the sum was delivered at the end of the 5-s period. **c**, Schematic representation of a knapsack trial when animal exceeded 0.8 ml. In this case, no reward was delivered and a 4-s timeout was imposed. **d**, An additional fractal image was introduced as a ‘positive control’ that promised a reward equal to the knapsack limit. Bar graphs show the percentage of positive control trials when the animals selected the positive control item and nothing else. This response is optimal. Orange and brown bar graphs show data from monkeys G and B, respectively. The gray bar graph shows the percentage of positive control trials that an agent using a random sampling strategy chooses the positive control image and nothing else. Error bars are ±s.e.m. across *n* = 791 and 620 trials for monkeys G and B, respectively. **e**, An additional fractal image was introduced as a ‘negative control’ that promised no reward. Box plots show the percentage of negative control trials when the animals exhibited optimal behavior, here defined as not including the negative control item in the solutions. Orange and brown box plots show data from monkeys B and G, respectively. The gray dotted line shows the percentage of negative control trials that an agent using a random sampling strategy avoids including the negative control item. *n* = 36 and 26 sessions for monkeys G and B, respectively. Box plots show the median (line), quartiles (boxes), range (whiskers) and outliers (+).

**Extended Data Fig. 1:**
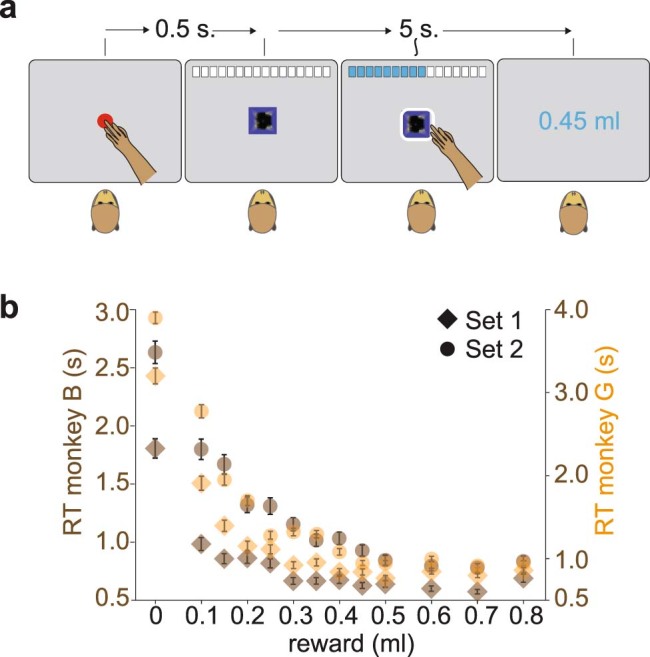
Item training. **a)** Schematic of item training task. The animals used a simple stimulus-response-reward task to learn the predictive value of each individual item in the stimulus set. **b)** Scatter plot of response times vs reward magnitude for monkey G (orange, left y-axis) and monkey B (brown, right y axis). Diamonds represent data from stimulus set 1 and circles represent data from stimulus set 2. Lower response times for large-reward cues indicated that the animals had learned the values. Error bars are SEM across trials for individual reward magnitudes. Monkey G experienced in total n = 5479 and n = 7151 trials for Set 1 and 2. Monkey B experienced in total and n = 6846 and n = 5379 trials for Set 1 and 2.

We trained two rhesus macaque monkeys to associate rewards with virtual items symbolized by fractal images displayed on a touchscreen computer. Eleven different items predicted reward sizes between 0.1 ml and 0.7 ml (Fig. 1a, left column), and the animals learned individual items’ values one at a time (Extended Data Fig. 1a). Once the learning criteria were achieved (Extended Data Fig. 1b), the animals performed the knapsack task within the 11 items. After approximately 1 month of working with the 11 items in the knapsack task, we discarded the trained fractal cues and trained the animals on a second set of fractal images that predicted the same reward values and repeated knapsack testing (Fig. 1a and Extended Data Fig. 1a,b). By using two stimulus sets for each animal, we ensured that the behavior was not dependent on specific sensory features of the cues.

The objective of the knapsack task was to select combinations of items whose sum maximized juice reward without exceeding a limit of 0.8 ml (Fig. 1b). Each knapsack trial began with the appearance of an initiation target. When the animal touched the initiation target, the screen displayed an ‘instance’, which was a combination of five distinct, randomly selected items. The touchscreen displayed all five items simultaneously, at pseudorandomized screen locations. When the animal selected an item by touching it, the item was highlighted and a virtual knapsack at the top of the screen was ‘filled’ by an amount equivalent to the item’s associated reward. Items could not be deselected. The instance remained on the screen for 5 s, and then, if the sum of the selected items was less than or equal to 0.8 ml, the animals were rewarded with the juice equivalent of the sum of the selected items (Fig. 1b). If, however, the animals selected items with a sum that exceeds 0.8 ml, no reward was delivered, and a 4-s timeout was added before the intertrial interval (Fig. 1c). Thus, the knapsack task incentivized the subjects to search for and select the optimal combinations.

In the context of the task, optimal solutions returned the largest reward with the fewest actions. To examine whether the animals sought to optimize, we introduced positive and negative control items that predicted 0.8 ml and 0.0 ml, respectively, and that enabled precise definitions of optimal behaviors (Fig. 1d,e, top). When the positive control item was part of a five-item instance, the optimal solution included the 0.8 item and nothing else. Both animals achieved this normative optimality on most positive control trials (Fig. 1d, bottom). The negative control item is all cost with no associated reward, and therefore the optimal solutions never included that item. The animals achieved this normative optimality in more than 95% of negative control trials (Fig. 1e, bottom). These results demonstrate that, when optimal behaviors are simple and objectively defined, the animals sought the optimal solutions.

**Fig. 2:**
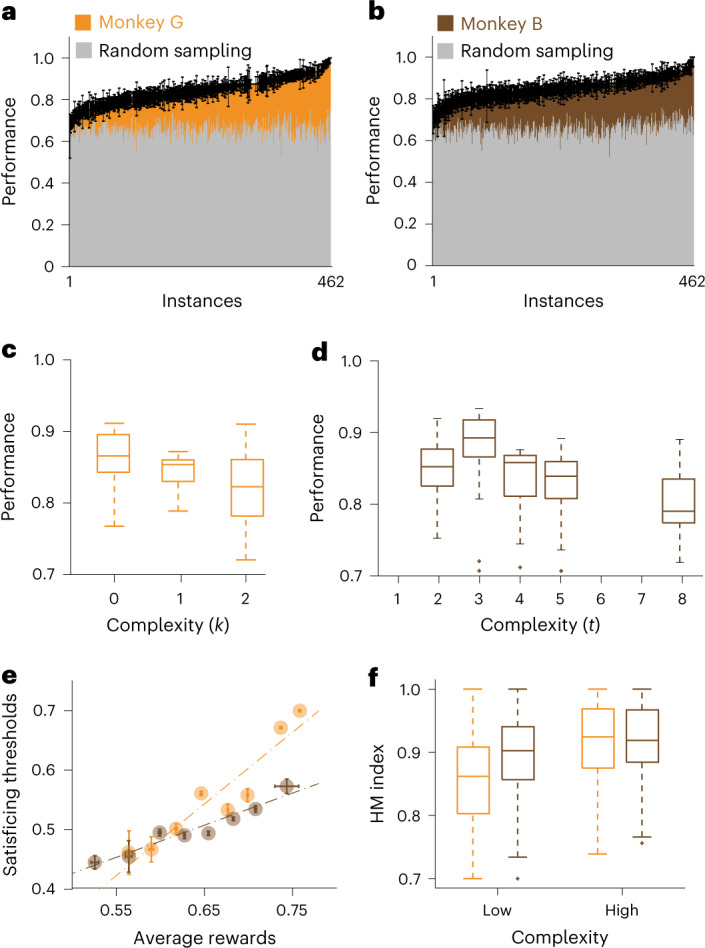
Instance complexity drives performance variability and promotes satisficing behavior. **a**, Bar graphs showing performance for all 462 instances, sorted according to performance. Behavioral performance on a single trial was defined as the size of juice reward the animal received on that trial, divided by the largest possible reward size available on that trial. Orange bar graphs show the performance of monkey G. Error bars are ±s.e.m. across on average *n* = 24 trials of the same instance. Superimposed gray bar graphs show the average performance of an agent using random sampling to generate solutions. **b**, As in **a**, for monkey B (brown). **c**, Box plots showing the session-averaged performance of monkey G as a function of instance complexity as measured by the clustering parameter, *k*. *n* = 36 sessions. **d**, Box plots showing the session-averaged performance of monkey B as a function of instance complexity as measured by the thresholding parameter, *t*. *n* = 26 sessions. **e**, Scatter plot showing the relationship between averaged rewards and satisficing thresholds. Horizontal error bars are s.e.m. across average reward earned in the binned instances. Vertical error bars are s.e.m. across satisficing thresholds for the binned instances (Methods). Orange and brown dots represent results from monkeys G and B, respectively. Orange and brown dashed lines are the best-fitting lines to data from monkeys G and B, respectively. The statistics in the main text are calculated using all *n* = 462 instances. **f**, Box plots showing the Houtman–Maks (HM) indices—quality of fit metrics for the satisficing model—for each instance separated according to instance complexity. Low-complexity trials include all trials with *k* = 0, whereas high-complexity trials have *k* > 0. Orange and brown box plots correspond to data from monkeys G and B, respectively. *n* = 462 instances. Box plots show the median (line), quartiles (boxes), range (whiskers) and outliers (+).

**Extended Data Fig. 2:**
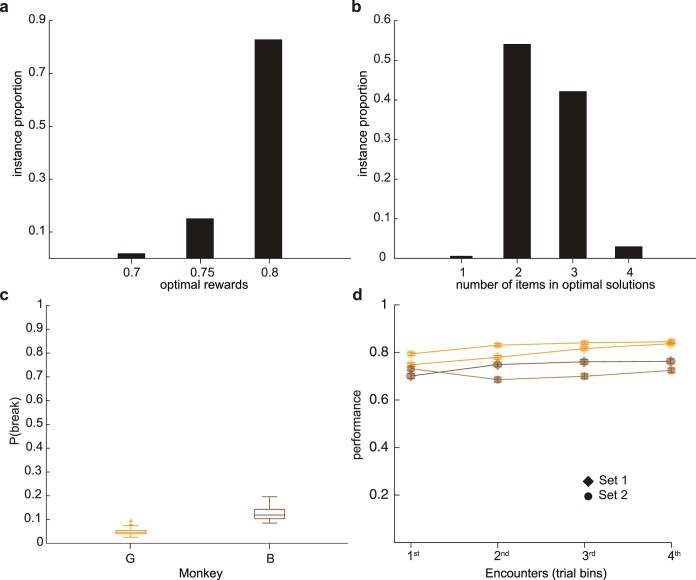
Summary statistics for the knapsack task and monkeys’ performance. **a)** Histogram shows the distribution of the sum of optimal solutions in the knapsack task. **b)** Histogram shows the distribution of the number of items included in the optimal solutions. **c)** Box plots show the probability monkey G (orange) and monkey B (brown) selected combinations whose sums exceeded the limit. n = 36 and n = 26 sessions for monkeys G and B respectively. **d)** Line plot shows the instance-averaged performance as a function of the number of repetitions the animals experienced. For each instance, both monkey G (orange) and monkey B (brown) experienced around 15 repetitions across a month for a single stimulus set. The repetitions for an instance were sorted according to the order the animals experienced them and binned into 4 bins. Diamonds are data from stimulus set 1 and circles are data from data set 2. Error bars are SEM across n = 462 instances.

When the positive and negative control items were not part of the instance and the optimal solutions were not readily estimated, the deliberative nature of the animals’ behavior was frankly evident in video recordings of the animal doing the task (Supplementary Video 1). For all 462 five-item combinations, we calculated optimal solutions and the number of items required to achieve the optimal solutions (Extended Data Fig. 2a,b). Behavioral performance on a single trial was defined as the size of the juice reward the animal received on that trial, divided by the largest possible reward size available on that trial. In nearly every instance, the animals’ performance exceeded levels that would be achieved by random sampling (Fig. 2a,b), and the probabilities of exceeding the limit and breaking the knapsack were uniformly low (Extended Data Fig. 2c). The performances on the first stimulus set were correlated with their performances on the second stimulus set (rho = 0.70 and rho = 0.25, *P* < 10^−69^ and *P* < 10^−8^, Spearman’s correlation). In fact, the animals achieved high-performance levels the first time they encountered instances and exhibited only minimal improvement over repeated exposures (Extended Data Fig. 2d). Together, these results indicated the following: (1) the animals achieved better results than predicted by chance, (2) the performance was not dependent on the sensory properties of the items and (3) high-performance levels were not the result of combination learning.

### Computational complexity increased difficulty and promoted satisficing behavior

**Extended Data Fig. 3:**
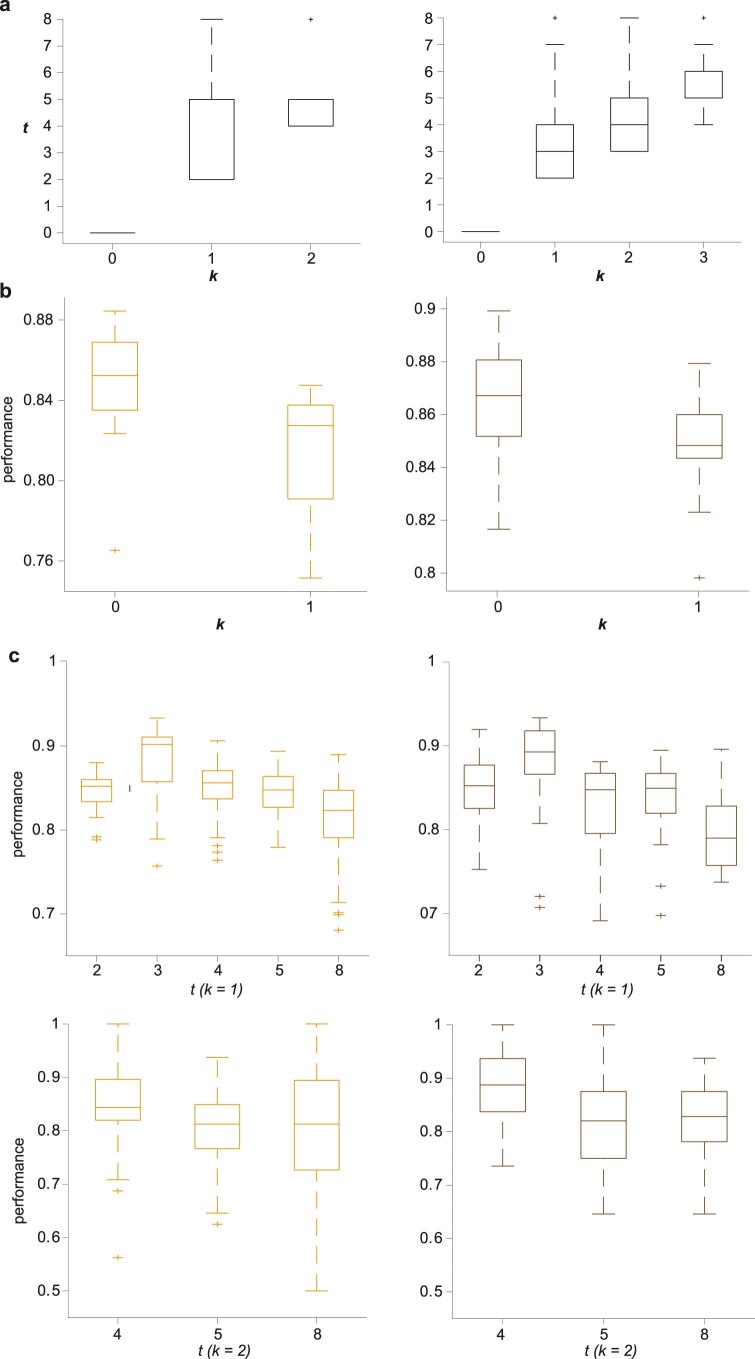
Performance was sensitive to both instance complexity measures. **a)** (left) Box plot shows the relationship between complexity defined by k and complexity defined by t under the range of values used in the task. The Spearman’s correlation between the two measures is 0.94. n = 462 instances. (right) Box plot shows the relationship between complexity defined by k and complexity defined by t under finer value intervals, ranging from 0.1 to 0.7 with a step of 0.01. The Spearman’s correlation between the two measures is 0.89. This finer scale of analysis is done simply to demonstrate that the significant correlation was not based on the values we used. n = 10^6^ instances **b)** Box plots show the relationship between monkey G’s (left) and monkey B’s (right) average session performance (excluding break trials) in the knapsack task with four items and complexity defined by k (p = 0.038 and 0.037, for monkeys G and B, respectively, rank sum test). Only one instance with complexity k = 2 exists for the four-item task and didn’t produce enough trials. n = 8 and n = 16 sessions for monkeys G and B respectively. **c)** Box plots show the average session performance (excluding break trials as a function of complexity defined by t (rho = −0.29, rho = −0.22, rho = −0.39, rho = −0.32 and p < 10^−4^, p = 0.036, p < 10^−5^, p = 0.01, for top left, bottom left, top right, bottom right, respectively, Spearman correlation). The top row restricts the analysis to instances with complexity k = 1, and the bottom row restricts the analysis to instances with complexity k = 2. Orange box plots show data for monkey G. Brown box plots show data for monkey B. n = 8 and n = 16 sessions for monkeys G and B respectively. Box plots show the median (line), quartiles (boxes), range (whiskers) and outliers (+).

In nearly all 462 instances, the animals performed better than predicted by chance, yet there was significant performance variability between instances (Fig. 2a,b). We sought to determine whether computational complexity modulated task difficulty and explained the performance variability. The complexity of the instances can be formalized as the computational requirements needed to find an optimal solution^4,10^. We parametrized the computational requirements using two algorithmic properties, combination size (*k*) and threshold (*t*). Although the specific algorithms that use *k* or *t* perform qualitatively different computations (Methods), the complexity measures derived from *k*- or *t*-based algorithms are highly correlated (Extended Data Fig. 3a). This result suggests that both measures approximated the intrinsic complexity in the knapsack task. Both animals achieved less optimal solutions as complexity increased (Extended Data Fig. 3b,c), regardless of the complexity measure. Monkey G was most sensitive to the combination size parameter (Fig. 2c; rho = −0.40, *P* < 10^−4^, Spearman’s correlation), whereas monkey B was most sensitive to threshold levels (Fig. 2d; rho = −0.20, *P* = 0.01, Spearman’s correlation). These results show that greater computational complexity resulted in lower performance, and strongly suggest that, in accordance with fundamental computational principles, greater computational complexity increased task difficulty.

**Extended Data Fig. 4:**
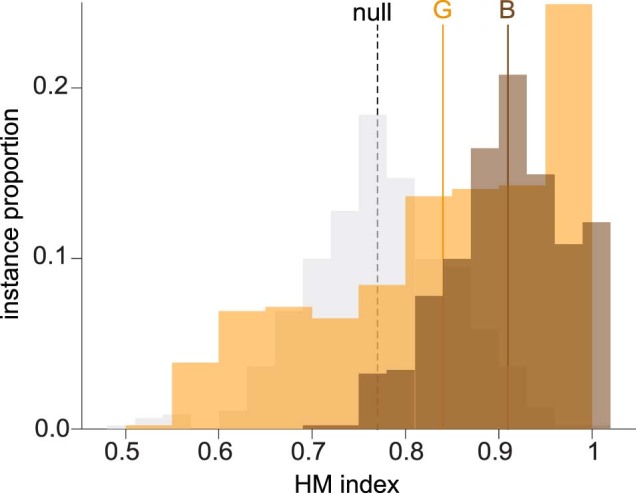
Both animals satisficed. Histogram shows distributions of HM indices across 462 instances. HM index calculates the proportion of selections in an instance that are consistent with the satisficing model (Methods). The null distributions (gray) were constructed by randomly sampling solutions that didn’t exceed the limit. The distributions for monkey G (orange) and monkey B (brown) were constructed using behavioral trials that didn’t exceed the limit. Both animals have significantly higher HM indices compared to the null distribution (p < 10^−18^ and p < 10^−72^, for monkeys G and B, respectively, sign rank test).

During complex deliberations, decision-makers ‘satisfice’, that is, decision-makers optimize to achieve satisfactory, rather than objectively, optimal outcomes^22^. We used an established model to determine the satisficing thresholds (Methods)^23^. For both animals, the estimated satisficing thresholds correlated positively with behavioral performances (Fig. 2e; rho = 0.528 and rho = 0.351; *P* < 10^−33^ and *P* < 10^−14^ for monkeys G and B, respectively, Spearman’s correlation). Critically, we applied the model independently to the behavior collected on stimulus sets 1 and 2 for both animals. The satisficing thresholds estimated from stimulus set 1 predicted the satisficing thresholds estimated from stimulus set 2 (rho = 0.358 and rho = 0.138; *P* < 10^−35^ and *P* < 0.007 for monkeys G and B, respectively, Spearman’s correlation). Thus, satisficing behaviors were modulated by the mathematical properties of the instances, rather than the sensory properties of the cues. We used the Houtman–Maks (HM) index to evaluate the fit of the satisficing model (Methods and Extended Data Fig. 4). When the computational complexity was greater, the satisficing model produced a better fit to the behavior data (Fig. 2f; *P* < 10^−33^ and *P* < 10^−3^ for monkeys G and B, respectively). Together, these results demonstrated that animals optimized to achieve satisfactory outcomes and suggest that their tendency to satisfice was dependent on the computational difficulty of the task.

### Efficient algorithms match behavioral solutions and predict deliberation times

**Fig. 3:**
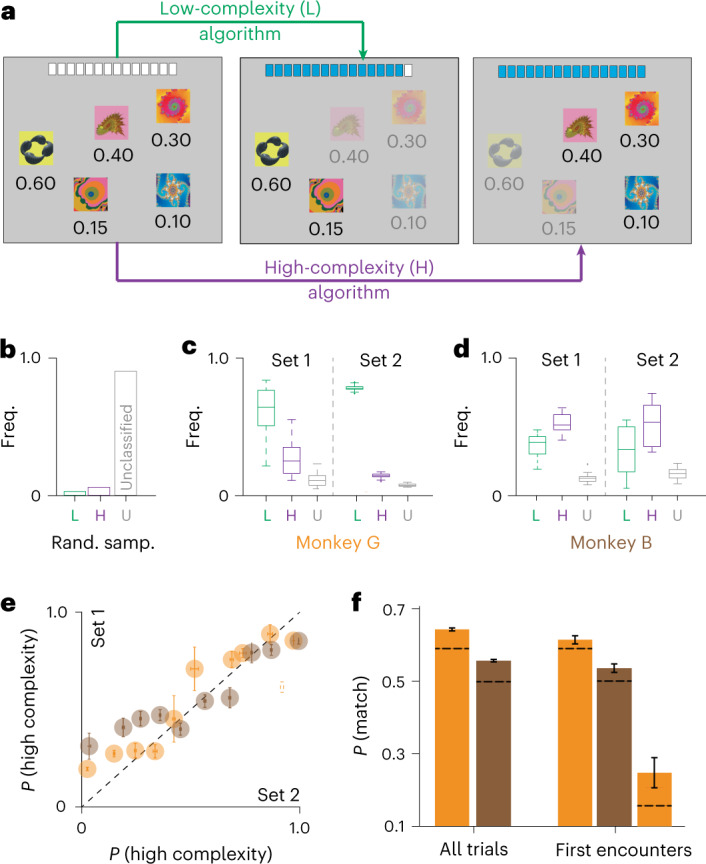
Behavioral solutions reflect distinct algorithmic reasoning strategies. **a**, Image showing different solutions to the same knapsack instance. The solution in the middle panel can be achieved by a low-complexity algorithm (green arrow), whereas the solution shown on the right can be achieved with a high-complexity algorithm (purple arrow). **b**, Bar graphs showing the distribution of possible classifications, low complexity (L), high complexity (H) or unclassified (U) for all possible solutions generated via random sampling. Note that most randomly sampled solutions remain unclassified (U). Classification abbreviations L, H and U apply to **b**, **c** and **d**, respectively. **c**, Box plots showing the distribution of classifications for solutions generated by monkey G in stimulus sets 1 and 2. *n* = 36 sessions. **d**, As in **c**, for monkey B. *n* = 26 sessions. **e**, Scatter plots showing the probabilities of classifying trials as high complexity for the same instances in stimulus sets 1 and 2. Horizontal error bars correspond to the s.e.m. across binned instances, for set 2. Vertical error bars correspond to the s.e.m. across binned instances, for set 1 (Methods). The statistics in the main text are calculated using all *n* = 462 instances. **f**, Bar graphs showing the probabilities that two randomly selected trials of the same instance (left), or the first encounter of the same instance in sets 1 and 2 (right), matched to the same algorithm. Orange and brown bar graphs represent data from monkeys G and B, respectively. Dashed black lines indicate chance levels, calculated as the probabilities that two trials from different instances are both of low and high complexity. Error bars are s.e.m. across *n* = 462 instances. Monkey G approximated low-complexity algorithms on a high percentage of trials, as can be seen in **b**; therefore the chance level of matching two trials from two different instances was high. As a result, the match between first encounters in sets 1 and 2 was not significantly different from chance, when we considered both low- and high-complexity trials (*P* = 0.06, binomial test). However, in instances where the animal applied a high-complexity algorithm on the first encounters in set 1, the probability that the first encounter on set 2 was high complexity was significantly above chance (*P* = 0.006, binomial test, the rightmost bar).

**Extended Data Fig. 5:**
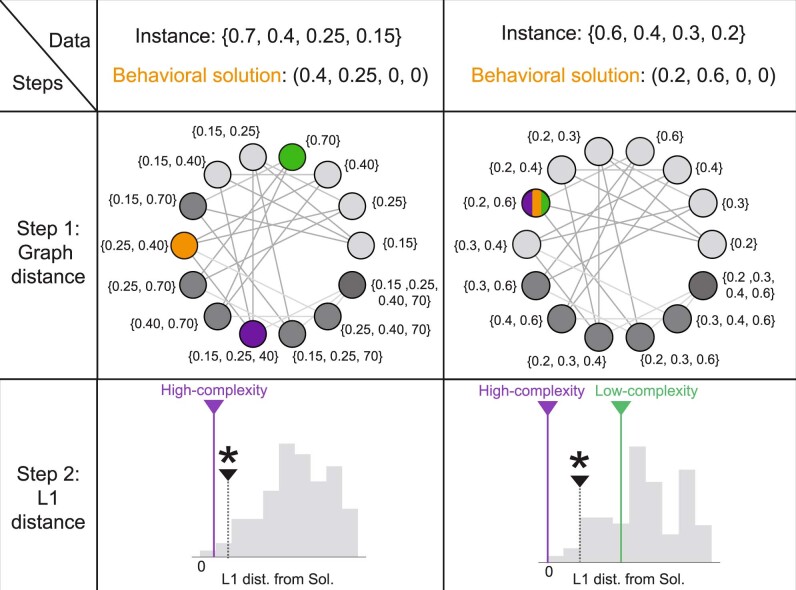
Trial classification procedure. The table provided two toy examples demonstrating how the two-step procedure worked. For the first step, the orange node in the undirect graph corresponds to the behavioral solution; the green node in the undirected graph corresponds to the solution generated by a low-complexity algorithm (greedy); the purple node in the undirected graph corresponds to the solution generated by a high-complexity algorithm (Sahni-1). For the second step, the gray histogram represents the null distribution constructed by random sampling and asterisk represents the lower 5% threshold. See Methods for more details on the classification procedure.

Different submitted solutions to the same instance might indicate that different reasoning strategies, or algorithms, are used to solve the same problem (Fig. 3a). We used a two-step procedure to classify the behavioral solutions as most likely to be generated by low-complexity algorithms that operate on items one at a time (*k* = 0 or *t* ≤ 1), or by high- complexity algorithms that examine combinations (*k* > 0 or *t* > 1) (Extended Data Fig. 5a; Methods). The procedure was strict—solutions derived by random sampling rarely met our criteria for classification and the vast majority remained ‘unclassified’ (Fig. 3b). This two-step procedure classified 91.5% and 86.3% of trials in monkeys G and B, respectively, as generated by low- or high-complexity algorithms (Fig. 3c,d). In fact, more than 40% of behavioral solutions exactly matched one of the algorithmic solutions, although the chance of randomly sampled solutions exactly matching any of the algorithmic solutions was less than 2.5%. Moreover, the matched algorithms were consistent between instances in using stimulus sets 1 and 2 (Fig. 3c,d). On an instance-by-instance basis, the algorithmic parameters that matched the behavior on stimulus set 1 were highly correlated with the parameters that matched the behavior on stimulus set 2 (Fig. 3e; rho = 0.51 and rho = 0.63; *P* = 10^−31^ and *P* = 10^−52^ for monkeys G and B, respectively, Spearman’s correlation), and the first encounter of each instance in stimulus sets 1 and 2 was matched far more often than predicted by chance (Fig. 3f). These results demonstrate that the animals’ strategies for optimizing rewards were neither random nor dependent on specific sensory features of the cues. Rather, the solutions reflected intentional application of reasoning strategies that approximated algorithms specifically designed to manage complex optimization problems.

**Fig. 4:**
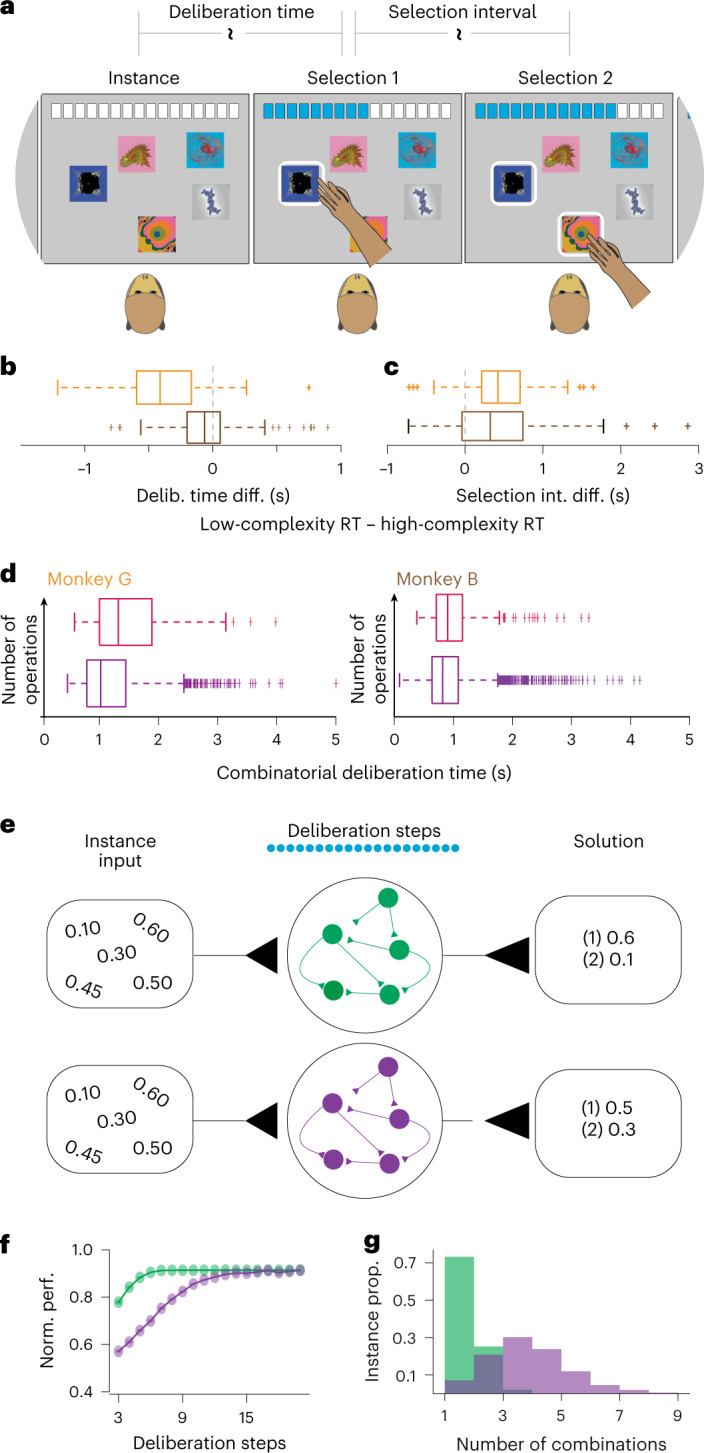
Deliberation times reflected algorithm requirements. **a**, Detail of the task schematic highlights the ‘deliberation time’, between the instance presentation and the first selection, and the ‘selection interval time,’ between the subsequent selections. **b**, Box plots showing the within-instance differences in average deliberation times between solutions classified as low or high complexity. Orange and brown box plots represent data from monkeys G and B, respectively. *n* = 201 and *n* = 270 instances for monkeys G and B, respectively. **c**, Box plots showing the within-instance differences in average selection intervals times between solutions classified as low or high complexity. Orange and brown box plots indicate data from monkeys G and B, respectively. *n* = 186 and *n* = 164 instances for monkeys G and B, respectively. **d**, Box plots showing the deliberation times for high-complexity solutions, divided according to whether the estimated thresholding parameter, *t*, successfully reduced (deep purple) or failed to reduce (magenta) the size of the combinatorial search space (Methods). *n* = 2507 and *n* = 5165 trials for monkeys G and B, respectively. **e**, Image of a training strategy for creating RNNs that simulated low-complexity (green) or high-complexity (purple) algorithms. The blue circles represent the 20 deliberation steps, and the target solutions are defined by the low- or high-complexity algorithms. **f**, Scatter plot for normalized performance when the networks were prematurely stopped during the deliberation steps. Normalized performance was the performance of the RNNs at each time step divided by the performance of the RNNs that underwent the entire deliberation period. **g**, Histograms show the frequency of considering different numbers of unique combinations during the deliberation steps. Green and purple histograms represent data from RNNs trained to mimic low- and high-complexity algorithms, respectively. *n* = 462 instances. Box plots show the median (line), quartiles (boxes), range (whiskers) and outliers (+).

A crucial difference between algorithms is the number and order of operations required to determine the selections. Before the first selection, low-complexity algorithms only need to identify the most valuable items, whereas high-complexity algorithms need more operations to identify the most valuable combinations. On the other hand, before the second selection, the low-complexity algorithm needs to identify the next most valuable item that fits in the knapsack, whereas the high-complexity algorithm has already identified the second selection as part of the initial combination. Accordingly, solutions classified as low complexity should be accompanied by shorter RTs during the deliberation phase and longer RTs during the selection interval, compared to the RTs in the deliberation phase and selection intervals, respectively, on trials classified as high complexity (Fig. 4a). This is indeed what we observed. The animals spent significantly more time during deliberation (Fig. 4b; *P* < 10^−29^ and *P* < 10^−5^ for monkeys G and B, respectively, signed-rank test, and Supplementary Videos 2 and 3), and less time between the subsequent selections (Fig. 4c; *P* < 10^−25^ and *P* < 10^−12^ for monkeys G and B, respectively, signed-rank test), on trials that generated high-complexity, compared to low-complexity, solutions. Because this comparison was limited to trials of the same instances, the deliberation time differences cannot be explained by external factors, such as instance properties, but rather highlight complexity differences between the internal reasoning processes. Furthermore, when we divided high-complexity solution trials according to the estimated size of the combinatorial search space (Methods), the deliberation times associated with limited searches were significantly shortened, compared to trials where all items were taken into consideration (Fig. 4d; *P* < 10^−6^ and *P* = 0.01 for monkeys G and B, respectively, linear regression). Together, these results provide strong corroboration for the classification of solutions and demonstrate that deliberation times reflected the complexity of the underlying mental process.

**Extended Data Fig. 6:**
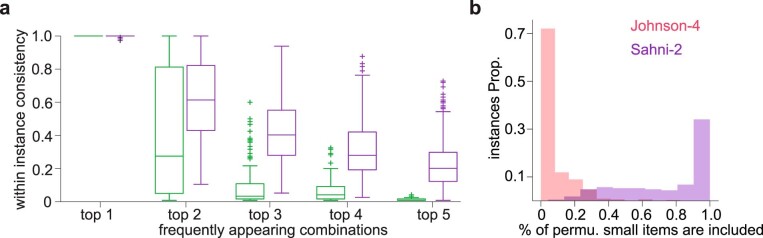
RNNs reflected the search process of their target algorithms. **a)** Box plot shows the within-instance consistency score for the top five most frequently appearing combinations for the low-complexity RNN (green) and the high-complexity RNN (purple). The most frequently appearing combinations for an instance were determined based on how often the combinations were represented across all time steps and permutations. The within-instance consistency score measured whether the combinations represented by the RNNs during deliberation were dependent on specific input order (Methods). A within-instance consistency of 1 means that the combinations are represented during deliberation no matter the order of the inputs. n = 462 instances for each box plot. Box plots show the median (line), quartiles (boxes), range (whiskers) and outliers (+). **b)** Histograms shows the proportion of permutations where the small items were present as part of the solutions generated under premature stopping for instances that actually contain the small items. The small items refer to items with a value lower than the threshold set by the Johnson-4 algorithm, which is 0.16. The purple distribution represents data from the Sahni-2 RNN and the pink distribution represents data from the Johnson-4 RNN.

The tight relationship between the deliberation times and the number of algorithmic operations suggests that computational algorithms are directly implemented by neural circuits in the brain. We, therefore, used recurrent neural networks (RNNs) simulating low- and high-complexity algorithms to investigate whether network implementations reflected the animals’ behavior (Methods). The networks deliberated and then generated ordered solutions (Fig. 4e; Methods). After training, we stopped the RNNs at different deliberation steps and forced the networks to generate solutions. Consistent with low-complexity behavioral solutions requiring less deliberation time, the low-complexity RNNs achieved target performance at earlier deliberation times, compared to the high-complexity RNNs (Fig. 4f). Correspondingly, the high-complexity network considered more unique combinations during deliberation, compared to the low-complexity RNN (Fig. 4g). Furthermore, the high-complexity network favored the same combinations across different permutations of the same instances, demonstrating that combinatorial deliberations were based on instance properties, rather than orders of the inputs (Extended Data Fig. 6a). Finally, RNNs that were trained to mimic different high-complexity algorithms, specifically those algorithms that used clustering (*k*) or thresholding (*t*) to limit the search space, reflected those different combinatorial strategies during deliberation (Extended Data Fig. 6b). These results demonstrate that neural network implementations are consistent with behavioral RTs and, moreover, that network-based deliberations resemble algorithm-specific reasoning strategies.

### Algorithm selection adapted to complexity

**Fig. 5:**
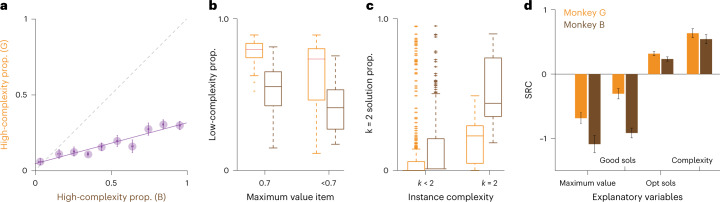
Algorithm selection adapted to complexity. **a**, Scatter plot of the proportion of solutions consistent with high-complexity algorithms, for matched instances in monkeys G and B. Horizontal error bars are s.e.m. across instances within bins, for monkey B. Vertical error bars are s.e.m. across instances within bins, for monkey G (Methods). Solid and dashed lines are the best fitting and unity lines, respectively. The statistic in the main text is calculated using all *n* = 462 instances. **b**, Box plots showing the proportion of low-complexity solutions instances when the maximum value item was 0.7 ml or less than 0.7 ml. Orange and brown boxes show data from monkeys G and B, respectively. *n* = 462 instances. **c**, Box plots showing the proportion of solutions that were consistent with a *k* = 2 algorithm under instances with complexity *k* < 2 or *k* = 2. Orange and brown bars show data from monkeys G and B, respectively. *n* = 462 instances. **d**, Bar plots showing the standardized regression coefficients (SRC) of independent variables reported in the text. Error bars are standard errors derived from the mixed-effects logistic regression model. *n* = 11511 and *n* = 8507 trials for monkeys G and B, respectively. Box plots showing the median (line), quartiles (boxes), range (whiskers) and outliers (+).

**Extended Data Fig. 7:**
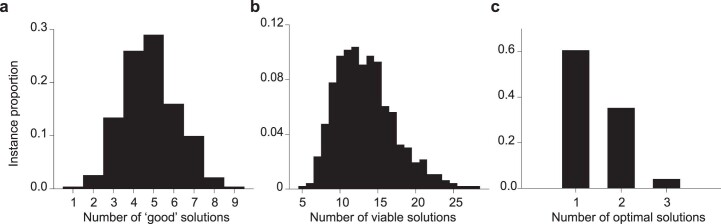
Summary statistics for variables in the logistic regression model. **a)** Histogram shows the distribution of the number of ‘good’ solutions in the knapsack task. **b)** Histogram shows the distribution of the number of viable solutions. **c)** Histogram shows the distribution of the number of optimal solutions.

Given the evidence that the animals approximate distinct algorithms for optimization, a fundamental question is—What factors determine algorithm selection? Although both animals used two stimulus sets (Fig. 3), the probabilities of approximating low- or high-complexity algorithms on given instances were highly correlated between the animals (Fig. 5a; *P* = 0.43 and *P* < 10^−22^, Spearman’s correlation). This result suggested that instance properties influenced algorithm selection in a predictable way. Indeed, the largest reward items promoted low-complexity strategies (Fig. 5b; *P* = 0.003 and *P* < 10^−24^ for monkeys G and B, respectively, rank-sum test). In contrast, the instances with the highest complexity, where *k* = 2, promoted the approximation of *k* = 2 behavioral strategies (Fig. 5c; *P* < 10^−4^ and *P* < 10^−7^, for monkeys G and B, respectively, rank-sum test). We applied logistic regression models to examine the influence of the full ranges of item values and instance complexities (Supplementary Tables 1 and 2; Methods). Consistent with the results for the largest reward and the highest complexity, both animals were more likely to use a low-complexity strategy as the maximum value item increased (Fig. 5d; *β* = −0.686 and *β* = −1.094, *P* < 10^−15^ and *P* < 10^−15^ for monkeys G and B, respectively), and both animals adapted to greater instance complexity by selecting high-complexity reasoning strategies (Fig. 5d; *β* = 0.638 and *β* = 0.547, *P* < 10^−20^ and *P* < 10^−13^ for monkeys G and B, respectively). For the number of viable solutions, we controlled the number of good solutions and the number of optimal solutions—factors that can influence human behavior (Extended Data Fig. 7)^4^. Interestingly, the number of ‘good’ solutions was positively related to selecting low-complexity strategies (Fig. 5d; *β* = −0.305 and *β* = −0.913, *P* = 10^−4^ and *P* < 10^−32^ for monkeys G and B, respectively), whereas the number of optimal solutions exerted the opposite effect (Fig. 5d; *β* = 0.318 and *β* = 0.239, *P* < 10^−22^ and *P* < 10^−11^ for monkeys G and B, respectively). The number of viable solutions oppositely affected the two animals, promoting low-complexity reasoning in monkey G and high-complexity reasoning in monkey B (Supplementary Tables 1 and 2). We also considered factors that could explain trial-by-trial variation, including the reward magnitude received in the previous trial, whether the animals exceeded the limit in the previous trial and the total amount of juice the animal consumed in the session. However, this model produced worse Bayesian information criterion (BIC) values for both animals, compared to the model with only instance-level factors (Supplementary Tables 1 and 2; Methods). Together, these results demonstrate general and subject-specific influences on metacognitive processing aimed at balancing mental efforts and optimal rewards.

## Discussion

Economic deliberation often requires sophisticated reasoning. Combinations of different commodities or different choice parameters, including gains, losses, uncertainty, time and effort, make utility maximization a computationally complex problem. Here we created the knapsack task to evoke temporally extended economic deliberations and used it to demonstrate that rhesus monkeys used algorithm-based reasoning to maximize rewards in a complex optimization task. The conclusion that the animals’ behavior reflected algorithmically driven combinatorial reasoning was based on the following five lines of evidence: (1) combinatorial difficulty of the instances affected the performance and satisficing behaviors (Fig. 2). (2) The behavioral solutions matched the order and identity of items selected by distinct computational algorithms (Fig. 3a,b). (3) The algorithms employed on particular instances were highly correlated between distinct stimulus sets and even across the animals, suggesting that ‘algorithm selection’ was neither random, nor based on the sensory properties of the stimuli, but based on the properties of the instance (Figs. 3c–e and 5a). (4) The deliberation times and subsequent RTs all reflected the number of operations each algorithm required at each time point (Fig. 4a–e). (5) The combinatorial difficulty predicted algorithm selection (Fig. 5). Together, these five lines of evidence overwhelmingly support the notion that rhesus monkeys deliberate over hard economic problems and employ algorithmic-based reasoning to optimize rewards. Together, these data establish a novel behavioral and computational framework for understanding the neural circuit basis for sophisticated economic reasoning specifically, and complex problem solving in general.

A fundamental aspect of optimization behavior is to determine what parameters are being optimized. In economic deliberation tasks, animals attempt to maximize utility by optimizing the cost–benefit tradeoffs^24^. In the context of the knapsack task, rewards provide utility, whereas actions and mental effort are costs that discount reward utility. Thus, the animals should seek the largest rewards while minimizing mental effort and actions. In knapsack trials that included the positive and negative control items, these cost–benefit tradeoffs, and therefore the optimal behaviors, were easily defined. The behavioral data from those trials indicated that the animals were optimizing economic value—they sought the largest possible reward with the fewest possible actions (Fig. 1d,e). However, as the problems become more complex, estimating utilities, action and mental effort costs became more difficult. On the benefit side of the equation, previous measurements of NHP utility functions have consistently shown that the knapsack limit we chose, 0.8 ml, falls on a roughly linear region of the utility function^25^. This was intentional; the decreasing marginal utility associated with large reward magnitudes could diminish motivation to find optimal solutions. Furthermore, because the utility is approximately linear in the ranges we used, reward size is an acceptable proxy for utility. On the other side of the cost–benefit equation, the notable costs incurred by the animals appeared to be associated with mental effort. As the complexity of the instances increased, the animals were more likely to use computationally demanding algorithms (Fig. 5c,d) and spend more time deliberating (Fig. 4a–d). Despite the increased mental effort, the performance of the animals declined as complexity increased (Fig. 2c,d and Extended Data Fig. 3). The inverse relationship between performance and algorithmic measures of complexity reflected that human performance data calibrated against a novel, algorithm-independent measure of complexity based on the mathematical structure of the problem^5^. Together, these results demonstrate that mental effort costs increase with computational complexity and highlight the fundamental compatibility of psychological reasoning with the normative principle that computational complexity increases computational requirements.

As the computational complexity and the associated mental effort increased, the animals appeared to ‘satisfice’. ‘Satisficing’ is a bedrock principle of human decision making; it is defined as finding solutions that are subjectively satisfactory and sufficient^22^. Here we observed robust evidence that the animals applied a satisficing threshold during knapsack behavior (Fig. 2e). Applying satisficing thresholds, rather than seeking objectively optimal rewards, does not contradict the evidence that the animals were applying optimization procedures. Rather, it demonstrates that the animals approximated combinatorial optimization algorithms to achieve subjectively optimal outcomes. Thus, our data suggest that computational complexity and greater mental efforts were needed to improve the outcomes in highly complex instances influenced by satisficing thresholds. A fundamental question to ask, therefore, is—What determines satisficing thresholds? Unfortunately, the current experiment did not collect choice process data^23^—data that could reveal the step-by-step search process. Therefore, it is beyond the scope of the current data to reveal the psychological mechanisms that determine satisficing thresholds. Future studies that include high-resolution eye-tracking and neural recording data can provide more complete pictures of how the animals conduct combinatorial searches and what factors influence satisficing thresholds. We suspect that understanding the neural mechanisms for determining a satisfactory reward size will be a crucial component for a complete understanding of economic behaviors.

To promote the generalizability of the algorithmic analysis of reasoning strategies, we chose to focus on elementary and psychologically plausible algorithms that employ common algorithmic parameters, including a clustering parameter *k* and a threshold parameter *t*. Both parameters manage the size of the search space; the clustering parameter determines the size of combinations to consider, whereas the *t* reduces the search space by ignoring items that fall below a threshold^12,13^. A convenient feature of *k* and *t* is that when *k* = 0 or *t* ≤ 1, the search strategy is ‘greedy’—it considers items one at a time and always includes the largest available item that fits^11^. We refer to this condition, when *k* = 0 or *t* ≤ 1, as the ‘low-complexity’ algorithm. When *k* > 0 or *t* > 1, the search process considers combinations of items, rather than singletons. We refer to these as ‘high-complexity’ algorithms. Both *k* and *t* classified task complexity in similar ways (Extended Data Fig. 3a), and both parameters are used by large families of algorithms. These broad algorithm families include the greedy algorithm, the Sahni-*k* algorithms, algorithms that are variants of greedy and Sahni-*k*, the Johnson-*t* algorithms, and algorithms that apply dynamic programming on large value items^11–13,26–28^. Thus, the core reasoning strategies that the animals used are consistent with many specific algorithms.

From the perspective of algorithm identification, a potential concern is how to account for noise in the animals’ behavioral solutions. To minimize the misclassification of behavioral strategies, we developed a strict, two-step classification procedure using graph distance and L1 distance, as well as significance thresholds determined by instance-specific distributions of randomly selected solutions (Extended Data Fig. 5). We show that this two-step procedure is sufficient to classify approximately 90% of the animals’ solutions according to low- or high-complexity algorithms, while at the same time leaving approximately 90% of randomly sampled solutions as unclassified (Fig. 3b). Thus, this two-step method provides a robust framework for classifying the behavior of the animals. The deliberation and RTs, moreover, provide strong validation for our classification scheme. The number of operations used by the best-matching algorithms predicted the duration of deliberation and item selection (Fig. 4a–d). In short, the greater the number of operations, the longer the deliberation and RTs. Together, the classification procedure and RT data provide strong evidence that the animals flexibly used different algorithmic reasoning strategies to optimize rewards.

The logistic regression modeling revealed multiple instance-level factors that predicted whether low- or high-complexity algorithms would be applied to an instance. For example, instance-level properties, such as the presence of the largest reward items or high instance complexity, promoted low- and high-complexity algorithm selection, respectively. In contrast, trial-level factors, such as the size of the previous reward—whether the previous trial resulted in breaking the limit or the total amount of reward consumed in a session, had little to no influence on current trial algorithm selection. Thus, our data predict well the behavior of the animals at the level of the instance but leave unexplained trial-by-trial variation. This highlights a fundamental limitation of our study in comparison to previous studies on human decision-makers^4,5^. Our current data do not reveal to what degree the animals explored space for possible solutions. Human studies allowed participants to select, try and deselect items to optimize. This ability to deselect items provided a direct window in the trial-level search process. For reasons of simplicity, we did not allow the animals to deselect items. Moreover, because the data were recorded in freely moving animals in their home cages, we were not able to track pupil gaze data. Lacking deselection and gaze data, we do not have a direct measurement of the search process, and we expect that this lack of search process data partially accounts for our inability to explain trial-by-trial variation. Moving forward, we will record behavioral and neuronal data simultaneously in head-fixed animals. This arrangement will enable us to track saccadic eye movements that have the potential to reveal the combinatorial search process.

Appropriate decision making is crucial for survival, and therefore understanding the neural basis of deliberation and decision making is a priority of modern neuroscience. Studies of perceptual judgments have revealed a psychologically intuitive and neuroscientifically grounded mechanism for the deliberate considerations of sensory evidence leading to choices^29,30^. A key element to that research program, as well as the deep mechanistic insights that it has generated, is the normative control of perceptual difficulty. For example, low-coherence motion in a random-dot kinematogram is normatively harder to perceive than high-coherence motion. This normative scale is the keystone of studies revealing neuronal correlates of evidence integration and decision boundaries in the lateral intraparietal area^31^, caudate^32^ and prefrontal cortex^33^. Here we demonstrate that computational complexity constitutes a normative scale for modulating the behavioral difficulty of economic decisions in NHPs. Thus, the knapsack task provides a principled framework to investigate the neural basis for temporally extended, effortful economic deliberations.

Our ultimate goal is to understand how we make economic choices. As a general theory of real economic choices, pure utility maximization is computationally intractable. Thus, understanding how the brain manages computational complexity is fundamental to understanding economic decisions^6–8^. Here we demonstrated computational and algorithmic insights into complex economic deliberations, but the complexity was purposefully constrained at a level where the animals approximated rational reasoning and true utility maximization. Future studies using this task to increase complexity to computationally intractable levels, where logical reasoning must fail and adaptive heuristics are required^34^, will be equally critical to gain a complete understanding of decision making. Therefore, the current results and the behavioral framework that they establish are a foundation to investigate the neural embodiment of economic computation, logical reasoning and human-level insight.

## Methods

### Animals and experimental setup

All animal procedures were approved by the Institutional Animal Care and Use Committee of the University of Pittsburgh. For this study, two male rhesus macaque monkeys (*Macaca mulatta*), weighing 13.6 kg and 9.8 kg and aged 8 years and 6 years, respectively, were used. In-cage training system (Thomas RECORDING GmbH) was attached to the cage and the tasks were presented on a touchscreen Samsung tablet. During experiments, free-moving animals interacted with the tablet to complete the trials. Black currant juice was delivered by a tablet-controlled solenoid liquid valve. Custom-made software (Android) running on the tablet controlled the behavioral tasks.

### Behavioral training and knapsack task

We generated two stimulus sets, each containing 11 fractal images that predicted rewards between 0.1 ml and 0.7 ml (Fig. 1a). We trained freely moving monkeys (*n* = 2 male rhesus macaques) the predictive value of each item (fractal image) in the stimulus set. On each training trial, the animal touched a ‘self-initiation’ cue, and then one item was pseudorandomly selected from the set and presented on a touchscreen. When the animal touched the screen where the item was presented, a virtual knapsack, pictured above the item, was filled with the ‘volume’ associated with the item, and the animals were rewarded with that same reward volume (Extended Data Fig. 1a). Training for each stimulus set lasted for approximately 2 weeks. We used the animals’ RTs and skipping rates to measure learning progress. For the subsequent behavioral testing, we used one stimulus set for approximately 1 month in the knapsack paradigm. Then, we discarded the fractals and trained new ones. The average number of repetitions for instances in stimulus set 1 was 13.6 and 10.7 and for stimulus set 2 was 13.6 and 10.5, for monkeys G and B, respectively.

Each knapsack trial began with an initiation cue, and then the touchscreen displayed an ‘instance’—a combination of five items that appeared, simultaneously, on the screen (Fig. 1b). The binomial coefficient formula indicates that there are 462 five-item combinations. The animals were limited to 300–400 trials per day, and instances were chosen randomly. Thus, it was uncommon that the same instance was repeated on any 1 d. The animals used the touchscreen to select items one by one. Whenever an item was selected, the following two things happened: (1) the item was highlighted and (2) the virtual knapsack at the top of the screen was filled with an amount equivalent to the item’s predicted reward. The knapsack limit was set at 0.8 ml. This limit was chosen based on previous studies that demonstrated the reward utility functions were relatively linear around 0.8 ml^1,2^. Optimal performance on every trial was defined as the largest possible sum of rewards less than or equal to the limit. If the animals selected a combination of items whose reward magnitude sum was less than optimal, they were rewarded by that lesser amount. If they selected a combination that was greater than the limit, then the trial ended, no reward was delivered and there was a 4-s timeout.

### Instance complexity

A previous study has shown that the complexity of an instance can be quantified as the minimum level of *k* necessary to achieve optimality with the Sahni-*k* algorithm^4^. The parameter *k* scales with the time and memory requirement. Similar in spirit, we defined another complexity metric based on the minimum level of *t* necessary to achieve optimality with the Johnson-*t* algorithm. Like parameter *k*, the parameter *t* also scales with the time and memory requirement. A detailed description of the specific computations performed by the Sahni-*k* and the Johnson-*t* algorithm is given in the subsection Candidate algorithms. To assess the correlation between *k* and *t*, we simulated a million five-item instances with values randomly sampled from 0.1 to 0.7 with 0.01 interval (Extended Data Fig. 3a). Without loss of generality, the limit is set to be 0.8 ml. To assess the relationship between performance and instance complexity, we only considered trials where the animals did not exceed the limit.

### Satisficing behaviors

The satisficing condition in the context of the knapsack task is as follows: The animals were satisficed if, and only if, they stopped selecting when the accumulated sum met their internal thresholds and continued selecting new items when the accumulated sum was below the internal thresholds. For each instance, we estimated the internal threshold by applying a binary decision model from a previous study that tested satisficing behaviors in humans^23^. Here we restated the model formulation. Let *θ* be the internal threshold to estimate, and *v* be the accumulated reward so far. The decision maker stops if and only if *v* ≥ *θ* + *ε*, where *ε*~*N*(0,0.1). Hence, the probability of stopping adding items to the sack is Φ(*v*-*θ*), where Φ is the cumulative distribution of *N*(0,0.1). We used maximum likelihood to estimate *θ*. For instances where the animals only selected one item in all individual trials, the maximum likelihood procedure failed. In this case, we used the mode as the threshold. The HM index quantified how well this model explained the animals’ behaviors. It calculated the proportion of selections that was consistent with the satisficing condition under the estimated thresholds. The higher the HM index, the better the satisficing model described the animals’ behaviors. Given the knapsack limit, the animals often stopped adding items to the sack at some point to avoid breaking. Hence, the HM indices can be inflated. To address this issue, we also fitted the model to solutions generated by a random agent that only outputted solutions whose sums were below the limit and constructed a null distribution of HM indices (Extended Data Fig. 4).

In addition, we also calculated Spearman’s correlation between the average rewards for individual instances and their HM indices. For visualization purposes, the instance-averaged rewards were sorted from smallest to largest and divided into eight equally spaced bins (Fig. 2e).

### Candidate algorithms

The greedy algorithm picks the largest available item that fits the residual sack at each step. The Sahni-*k* algorithm searches among all k-item combinations and fills the residual sack using the greedy algorithm. As the last step, it compares all constructed solutions. The Johnson-*t* algorithm searches for the best combination among items with a value higher than $\frac{{{\mathrm{limit}}}}{{t + 1}}$ before filling the residual sack using the greedy algorithm. We considered the greedy algorithm as a low-complexity algorithm and the Sahni-*k* algorithm and Johnson-*t* algorithm as high-complexity algorithms for the following reason. Let *n* be the number of the items. The worst-case complexity for the greedy algorithm is ${{{\mathcal{O}}}}\left( {n{\mathrm{log}}\left( n \right)} \right)$, whereas the worst-case complexity for the Sahni-*k* algorithm and Johnson-*t* algorithm is ${{{\mathcal{O}}}}\left( {n^{k+1}} \right)$ and ${{{\mathcal{O}}}}\left( {n^t} \right)$, respectively, when *k* > 0 and *t* > 1. Although the dynamic programming algorithm solves the knapsack problem exactly, we did not consider it, because its required memory size is much larger than the capacity of human working memory^4^. Moreover, as the animals only experienced the juice reward on a continuum rather than discrete drops, dynamic programming–based methods are not applicable to our task.

### Matching algorithms to behavior solutions

For each trial, we first calculated the graph distance between each candidate algorithm’s solution and the animals’ solutions. We constructed an undirected graph, where the nodes of the graph represented all potential combinations. Two nodes are connected if one node can be reached from another by adding or removing a single item and the corresponding edge weight is the value of that item^8^. We defined the distance between any two combinations to be the length of the shortest path between the two representative nodes. The distance between any two nodes *n*_1_ and *n*_2_ can be computed as follows:

$$d_{{\mathrm{graph}}}\left( {n_1,n_2} \right) = \mathop {\sum }\limits_{e \in U - I} e$$

where ${\mathrm{union}}(U) - {\mathrm{intersection}}(I)$ refers to set subtraction between set *U* and *I*, and *e* refers to an element in the set subtraction. Note that for graph distance, the order of the items is not considered.

Among the algorithmic solutions that have the smallest graph distances to the behavioral solution, we then ranked the candidates according to L1 distance. Each solution was defined as an ordered tuple and padded with zeros. For example, if the animal selected 0.4 ml, then 0.2 ml, then 0.1 ml and then stopped, the solution was defined as 0.4, 0.2, 0.1, 0 and 0, respectively. Thus, the distance between two solutions *p* and *q* can be characterized by the L1 distance.

$$d_{l_1}\left( {p,q} \right) = \mathop {\sum }\limits_{i = 1}^5 |p_i - q_i|$$

As the high-complexity algorithms do not specify the selection order for the items considered during the combinatorial search, we ordered the items selected during the combinatorial search to yield the smallest L1 distance to the behavioral solution.

To minimize the possibility that all algorithms match the behavioral response poorly, we constructed a null distribution for each trial by calculating the L1 distance between all possible solutions ($$\mathop {\sum }\nolimits_{i = 1}^5 \left( {\begin{array}{*{20}{c}} 5 \\ i \end{array}} \right) \ast i!$$ = 325 in total) and the behavioral response. The best-matching algorithm has to have a smaller L1 distance to the behavioral solution than the lower 5% threshold of the null distribution. Trials, where this criterion was not met, were labeled as ‘unclassified’. This procedure described the behavioral solutions well and retained most of the trials (8.5% of all trials were discarded for animal G, and 13.7% of all trials were discarded for animal B). Two examples are provided in the Extended Data Fig. 5.

Using this procedure, we inferred the parameters *k* (*k* = 0, 1, 2, 3) and *t* (*t* = 0, 2, 3, 4) that best described the behavioral solution on each trial. In total, there were seven candidate algorithms: the greedy algorithm, the Sahni-1 algorithm, the Sahni-2 algorithm, the Sahni-3 algorithm, the Johnson-2 algorithm, the Johnson-3 algorithm and the Johnson-4 algorithm (*k* = 0 and *t* = 0,1 are equivalent to the greedy algorithm, which is of low complexity). In cases where more than one level of the same parameter survived the procedure, we assume that the animals used the simpler algorithm and assign the minimum level of *k* or *t* accordingly. If both *k* and *t* survived the procedure, we considered the trial to be consistent with both types of parameters instead of arbitrating between the two.

The algorithms that survived the procedure described the animals’ behaviors well. More than 40% of all trials achieved zero L1 distance, around 52% of all trials achieved an L1 distance less than or equal to 0.05 ml and around 75% of all trials achieved an L1 distance less than or equal to 0.1 ml.

### Comparisons between data from different stimulus sets or animals

To determine whether the animals applied the algorithms according to the mathematical properties of the instances, we leveraged the fact that two different stimulus sets were used during data collection. We assessed whether the animals applied the algorithms with the same frequency across two stimulus sets by separately calculating the proportion of high-complexity solution trials within instances for trials in stimulus set 1 and stimulus set 2 and computing Spearman’s correlation across instances. For visualization purposes, we first binned the instances into ten equally spaced intervals according to the frequency the animals used high-complexity algorithms in stimulus set 2. Within each bin, we calculated the same frequency over the same instances in stimulus set 1 (Fig. 3e). Similarly, we estimated the satisficing threshold for each instance using trials in stimulus set 1 and stimulus set 2 separately and computed Spearman’s correlation across instances.

We also tested whether both animals were influenced by the same instance properties by separately calculating the proportion of low-complexity solution trials within instances for monkeys G and B and computing Spearman’s correlation across instances. For visualization purposes, instances were sorted according to an increasing proportion of solutions consistent with high-complexity algorithms, for monkey B. We used the sorting order from monkey B to sort instances from monkey G. The instances were then divided into ten equally spaced bins (Fig. 5a).

### Deliberation time analysis

Deliberation time was defined to be the time between the appearance of the fractals and the animals’ first touch. Behavioral variability allowed us to compare deliberation time between the low- and high-complexity trials within the same instance. For instances where the animals exhibited behaviors consistent with both low- and high-complexity solutions (each group must have more than three trials), we calculated the difference between the average deliberation times of the corresponding trial types and tested whether this difference was significantly different from zero across available instances.

Among high-complexity trials, we further explored whether deliberation time was modulated by the size of the search space. The search space could be modified by a lower bound when there are items with a value lower than the threshold. For each trial, we derived the estimated lower bound from the best-matching parameter *t* and separated the high-complexity trials into two classes. The first class included trials where the thresholds were larger than the minimum values in the instances. When the thresholds were larger than the minimum value, they were ineffective. The second class included trials where the thresholds were lower than the minimum values in the instances. A linear model with random intercepts was used to perform the comparison as follows:

$${\mathrm{deliberation}}\,{\mathrm{time}} = \beta _0 + \beta _1 \ast {\mathrm{effective}}\,{\mathrm{threshold}} + \left( {1|{\mathrm{session}}} \right)$$

where effective threshold is a binary variable that equals 1 when the thresholds were successful at reducing the size of the search space, and 0 otherwise.

One prediction of the longer deliberation times observed in the high-complexity trials is that the animals should spend less time thinking about the subsequent selections (that is selections after the first one). To confirm this prediction, in each instance, we calculated the difference between the average selection times across the subsequent selection intervals of the corresponding trial types. This analysis confined us to consider the trials where the animals chose more than one item.

### RNNs configurations and training

We trained RNNs to mimic low-complexity (greedy) and high-complexity (Sahni-2) algorithms using the PyTorch machine learning framework. The task had two distinct periods—deliberation and selection. The networks were trained to only output a specific ‘hold’ token during the deliberation period and output target solutions during the selection period. Both networks were trained to deliberate for 20 time steps before selecting any items. The RNNs have 512 hidden LSTM units, seven input units and six output units. During the deliberation period, the first five input units received the constant item values, the sixth input units received the constant capacity of the sack and the seventh unit always received 0. During the selection period, the seventh input unit received an input of 1 to signify the selection period. To recreate the visual feedback during the behavioral trials, the rest of the inputs were modified according to the previous selections—the sixth input unit now represents the remaining capacity based on the previous selections, and one of the five input units turns 0 if the corresponding item is chosen. For each algorithm, we provided the RNN with all permutations of all 462 instances (55,440 permutations in total) and their corresponding solutions as targets. For the Sahni-2 RNN, we randomly picked an order for the items selected by the corresponding algorithms during the combinatorial search.

Networks were initialized randomly, and maximum training epochs were set to 200. We used the Adam optimization algorithm and scheduled sampling with exponential decay (*ε* = 0.99^epoch^) to train the RNNs. To assess how well the RNNs imitate their targets, we defined the metric to be the indicator function that compares the solutions produced by the networks and the target one

$$I\left( {\left( {\mathop {\sum }\limits_{i = 1}^n I\left( {s_i = t_i} \right)} \right) = n} \right)$$

where *s*_*i*_ is the *i*th chosen item in the solution produced by the network and *t*_*i*_ is the *i*th chosen item in the target solution. We evaluated the accuracy using a held-out data set and confirmed that all RNNs achieved above 98% accuracy.

### Counting represented combinations during the deliberation period

To understand how the RNNs processed information during the deliberation period, we prematurely stopped the RNNs from deliberating and forced them to start selecting items. To do so, at a given time step during deliberation (ranging from the third time step to the 20th time step), we turned on the input that signifies the selection period. All RNNs immediately stopped outputting the ‘hold’ token and started selecting items. We examined the solutions produced by the prematurely stopped RNNs and measured performance as a function of the number of deliberation time steps. Moreover, we also analyzed how the composition of the solutions changed as a function of the number of deliberation time steps. Because the Sahni-2 algorithm performs a combinatorial search over pairs of items (referred to as ‘combination’ below) and selects the combination first when generating the solutions, we considered that a combination was represented during the deliberation period if it was present as the first two selections in the solutions generated under premature stopping. We counted the number of unique combinations that were represented during the deliberation period for the low- and high-complexity RNNs. To assess whether the combinations were robustly represented regardless of the order of the inputs, we computed the within-instance consistency. The within-instance consistency of a combination under an instance was the proportion of permutations where that combination was represented. We also examined whether the RNN reflected the search process of their algorithmic targets. In addition to the Sahni-2 RNN, we trained an RNN to mimic the Johnson-4 algorithm. Unlike the Sahni-2 algorithm, the Johnson-4 algorithm does not have a fixed-size search space, which makes it harder to examine how exactly did the solutions change by stopping the network prematurely. However, the defining signature of the Johnson-*t* algorithm is that items with a value smaller than a preset threshold are ignored during the combinatorial search. Hence, for each instance, we counted the proportion of permutations where items with a value less than 0.8/(4 + 1) = 0.16 ml were present in the solutions generated under premature stopping.

### Algorithm selections

To better understand the factors that modulated algorithm selection, we used a logistic regression model with random effects to predict whether the animals used low- or high-complexity algorithms on a trial-by-trial basis. The main variable of interest is instance complexity. We coded the instance complexity as a binary variable, which equals 0 when the low-complexity algorithm suffices to achieve optimality and equals 1 when the low-complexity algorithm fails. In addition, we included the value of the single fractals and defined four high-level properties of the instances that could affect the algorithm selection process. The number of viable solutions counts the number of solutions whose sums are below the limit.

$${\mathrm{number}}\,{\mathrm{of}}\,{\mathrm{viable}}\,{\mathrm{solutions}} = \mathop {\sum }\limits_S I\left( {\mathop {\sum }\limits_{x \in S} x \le {\mathrm{limit}}} \right)$$

where *S* is a solution of an instance.

We also assessed the optimality of a randomly behaving agent by calculating their average performance for each instance (random score). The random agent only selects solutions whose sums are below the limit:

$${\mathrm{random}}\,{\mathrm{score}} = \frac{1}{{|{{\Lambda }}|}}\mathop {\sum }\limits_{s \in {{\Lambda }}} \left( {\frac{{\mathop {\sum }\nolimits_{x \in S} x}}{{{\mathrm{opt}}}}} \right)$$

where

$${{\Lambda }} = \left\{ {{{{\mathrm{S}}}}:\mathop {\sum }\limits_{x \in S} x \le {{{\mathrm{limit}}}}} \right\}.$$

Furthermore, we quantified how easy it is to achieve a ‘good’ result by counting the number of solutions whose sums are above 0.6 ml juice. Mathematically, the property was defined as

$${\mathrm{number}}\,{\mathrm{of}}\,{\mathrm{good}}\,{\mathrm{solutions}} = \mathop {\sum }\limits_S I\left( {\tau _S \ge 0.6} \right)$$

where

$$\tau _S = \left\{ {\begin{array}{*{20}{l}} {\mathop {\sum}\limits_{x \in S} x ,} \hfill & {if\,\mathop {\sum}\limits_{x \in S} x \le {\mathrm{limit}}} \hfill \\ {0,} \hfill & {if\,\mathop {\sum}\limits_{x \in S} x > {\mathrm{limit}}} \hfill \end{array}} \right.$$

Similarly, the number of optimal solutions was defined as:

$${\mathrm{number}}\,{\mathrm{of}}\,{\mathrm{optimal}}\,{\mathrm{solutions}} = \mathop {\sum}\limits_S {I\left( {\tau _S = {\mathrm{opt}}} \right)}$$

In total, the model consisted of ten fixed effects. Because the animals performed hundreds of trials per session, we specified session-wise random effects. To determine the appropriate random effects, we considered the following model as the base model:

$$\displaystyle \log \left({\frac{{P\left( {{\mathrm{high}} \text{-} {\mathrm{complexity}}\,{\mathrm{solution}}} \right)}}{{1 - P\left( {{\mathrm{high}} \text{-} {\mathrm{complexity}}\,{\mathrm{solution}}} \right)}}} \right) = \beta _0 + \mathop {\sum}\limits_{i = 1}^{10} {\beta _i \ast x_i + \left( {1{{{\mathrm{|}}}}{\mathrm{sessions}}} \right)}$$

We then performed forward selection by including additional random slopes. For both monkeys, the forward selection process outputted the following model:

$$\begin{array}{l}\log \left(\displaystyle {\frac{{P\left( {{\mathrm{high}} \text{-} {\mathrm{complexity}}\,{\mathrm{solution}}} \right)}}{{1 - P\left( {{\mathrm{high}} \text{-} {\mathrm{complexity}}\,{\mathrm{solution}}} \right)}}} \right) = \\ \displaystyle \beta _0 + \mathop {\sum}\limits_{i = 1}^{10} {\beta _i \ast x_i + \left( {{\mathrm{max}}\,{\mathrm{value}} + {\mathrm{complexity}}{{{\mathrm{|}}}}{\mathrm{sessions}}} \right)} \end{array}$$

In addition to these instance-level factors, we also examined trial-level factors. We considered the reward magnitude received in the previous trial, whether the animals exceeded the limit in the previous trial, and the total amount of juice the animal consumed so far. This model produced worse BIC values for both animals, compared to the model with only instance-level factors (40,694 versus 40,623 for monkey B and 61,919 versus 61,856 for monkey G). We reported the statistics from the model with trial-level factors in Supplementary Tables 1 and 2.

### Statistics and reproducibility

All statistical analyses were performed, and all graphs were created in Python v.3.7.2 and MATLAB R2022a. No statistical methods were used to predetermine sample sizes, but our sample sizes are similar to those reported in studies investigating behavioral responses in NHPs^3,25,31,33^. All one-sample and two-sample statistical tests are nonparametric and two-sided. Wald test was used for testing the significance of coefficients in the regression models. Effects were considered significant at *P* < 0.05. No adjustments were made for multiple comparisons. Data collection and analysis were not performed blind to the conditions of the experiments.

### Reporting summary

Further information on research design is available in the Nature Portfolio Reporting Summary linked to this article.

## Online content

Any methods, additional references, Nature Portfolio reporting summaries, source data, extended data, supplementary information, acknowledgements, peer review information; details of author contributions and competing interests; and statements of data and code availability are available at 10.1038/s41593-023-01307-6.

## Supplementary information

Supplementary information Supplementary Table 1—Logistic regression model for algorithm selections (animal G). Supplementary Table 2—Logistic regression model for algorithm selections (animal B).

Reporting Summary

Supplementary Video 1 A nonhuman primate performs the knapsack task.

Supplementary Video 2 Example low-complexity solution trial.

Supplementary Video 3 Example high-complexity solution trial.

## Data Availability

The data from this study will be stored in a public repository maintained by the Open Science Framework. The repository can be found at https://osf.io/3d26g/.

## Code availability

The code used for the data analysis in this study is available online at https://osf.io/3d26g/.
