# Supplementary material for: Computational complexity drives sustained deliberation
Source: Nat Neurosci. 2023 Apr 24;26(5):850–7. doi: 10.1038/s41593-023-01307-6 (PMC10166852; doi:10.1038/s41593-023-01307-6)
Supplement: Supplementary file 1 — Supplementary Table 1—Logistic regression model for algorithm selections (animal G). Supplementary Table 2—Logistic regression model for algorithm selections (animal B). [file 41593_2023_1307_MOESM1_ESM.pdf]

nature neuroscience

Article

https://doi.org/10.1038/s41593-023-01307-6

Computational complexity drives sustained deliberation

In the format provided by the authors and unedited

Explanatory variables	SRC	SE	p value
Item 1	0.118	0.059	0.047
Item 2	0.137	0.067	0.040
Item 3	0.265	0.074	0.0003
Item 4	0.618	0.065	10^{-20}
Item 5	-0.686	0.087	10^{-14}
# of viable solutions	1.751	0.148	10^{-31}
Random score	0.005	0.093	0.957
# of ‘good’ solutions	-0.305	0.079	0.0001
# of optimal solutions	0.318	0.0324	10^{-21}
Instance complexity	0.638	0.069	10^{-19}
n-1 rewards	-0.016	0.052	0.754
n-1 break	-0.096	0.233	0.680
accumulated rewards	0.037	0.030	0.207
Intercept	-1.997	0.276	10^{-12}

Degrees of freedom	BIC
11497	62919

Table S1. Logistic regression model for algorithm selections (animal G). The table shows the results of estimation under mixed-effects logistic regression models with both instance-level and trial level variables for animal G. SRC: standardized regression coefficients; SE: standard error.

Explanatory variables	SRC	SE	p value
Item 1	-0.597	0.052	10^{-29}
Item 2	-0.857	0.060	10^{-44}
Item 3	-0.952	0.066	10^{-46}
Item 4	-0.766	0.054	10^{-43}
Item 5	-1.094	0.133	10^{-15}
# of viable solutions	-1.174	0.139	10^{-16}
Random score	1.284	0.083	10^{-52}
# of ‘good’ solutions	-0.913	0.076	10^{-32}
# of optimal solutions	0.239	0.033	10^{-12}
Instance complexity	0.547	0.071	10^{-13}
n-1 rewards	0.028	0.070	0.687
n-1 break	0.006	0.208	0.975
accumulated rewards	0.148	0.028	10^{-7}
Intercept	0.279	0.244	0.252

Degrees of freedom	BIC
8493	40694

Table S2. Logistic regression model for animal B. The table shows the results of estimation under mixed-effects logistic regression models with both instance-level and trial-level variables for animal B. SRC: standardized regression coefficients; SE: standard error.
